# Investigating the interplay of smoking, cardiovascular risk factors, and overall cardiovascular disease risk: NHANES analysis 2011–2018

**DOI:** 10.1186/s12872-024-03838-7

**Published:** 2024-04-04

**Authors:** Athumani Mambo, Yulu Yang, Emmerenceana Mahulu, Zhou Zihua

**Affiliations:** 1 Department of Cardiology, Union Hospital, Tongji Medical College, Huazhong University of Science and Technology, Wuhan, 430022 China; 2 Department of Cardiology, Benjamin Mkapa Hospital, P.O.Box 11088, Dodoma, Tanzania; 3 Department of Otorhinolaryngology, Benjamin Mkapa Hospital, P.O.Box 11088, Dodoma, Tanzania

**Keywords:** Cardiovascular disease, Smoking, Cardiovascular disease risk factors, Serum cotinine, Risk assessment

## Abstract

**Background:**

This study explores the intricate relationship between smoking, cardiovascular disease (CVD) risk factors and their combined impact on overall CVD risk, utilizing data from NHANES 2011–2018.

**Methods:**

Participants were categorized based on the presence of CVD, and we compared their demographic, social, and clinical characteristics. We utilized logistic regression models, receiver operating characteristics (ROC) analysis, and the chi-squared test to examine the associations between variables and CVD risk.

**Results:**

Significant differences in characteristics were observed between those with and without CVD. Serum cotinine levels exhibited a dose-dependent association with CVD risk. The highest quartile of cotinine levels corresponded to a 2.33-fold increase in risk. Smoking, especially in conjunction with lower HDL-c, significantly increases CVD risk. Combinations of smoking with hypertension, central obesity, diabetes, and elevated triglycerides also contributed to increased CVD risk. Waist-to-Height Ratio, Visceral Adiposity Index, A Body Shape Index, Conicity Index, Triglyceride-Glucose Index, Neutrophil, Mean platelet volume and Neutrophil to Lymphocyte ratio demonstrated significant associations with CVD risk, with varying levels of significance post-adjustment. When assessing the combined effect of smoking with multiple risk factors, a combination of smoking, central obesity, higher triglycerides, lower HDL-c, and hypertension presented the highest CVD risk, with an adjusted odds ratio of 14.18.

**Conclusion:**

Smoking, when combined with central obesity, higher triglycerides, lower HDL-c, and hypertension, presented the highest CVD risk, with an adjusted odds ratio of 14.18.

**Supplementary Information:**

The online version contains supplementary material available at 10.1186/s12872-024-03838-7.

## Introduction

Cardiovascular diseases (CVDs) remain a significant global health challenge with high morbidity and mortality rates [1, 2]. To effectively combat this burden, it is important to identify modifiable risk factors and understand their relationships with CVDs. Among these risk factors, smoking has long been recognized as a major behavioral contributor to CVD, with well-documented detrimental effects on cardiovascular health [3–5]. However, to gain comprehensive insights into the complex relationship between smoking, other cardiovascular risk factors, and overall CVD risk, up-to-date and comprehensive investigations are necessary.

Recent efforts to control tobacco use have led to declining smoking rates in some countries [6]. However, an investigation into the associations between smoking, traditional cardiovascular risk factors (e.g., obesity, dyslipidemia, hypertension and diabetes mellitus), and overall CVD risk, especially within the diverse U.S. population and the world in general, remains necessary. Given the substantial burden of CVD in the United States and beyond, understanding the relationship between smoking, cardiovascular risk factors, and overall CVD risk is crucial for effective prevention strategies [1–3, 5]. Despite existing research shedding light on the association between smoking and CVD risk factors, there remain several compelling reasons to conduct a comprehensive and current investigation.

Previous research efforts may have overlooked significant insights by not thoroughly exploring the full spectrum of risk factors, potentially missing important insights. The landscape of CVD risk factors and their associations is subject to evolution over time, driven by lifestyle changes, medical advancements, and shifting population characteristics [1, 7, 8]. Furthermore, the realm of tobacco products and patterns of tobacco use has undergone a substantial transformation, marked by the emergence of products like e-cigarettes and heat-not-burn devices [9, 10].

In tandem with these developments, recent progress in medical research has unveiled novel biomarkers and diagnostic tools capable of offering valuable insights into the early signs of cardiovascular damage linked to smoking [11]. Furthermore, the interaction between smoking, CVD risk factors, and emerging CVD risk indicators has not been extensively explored. While existing research has extensively investigated smoking’s impact on CVD risk factors [12–14], a research gap persists concerning how smoking combined with well-established CVD risks like central obesity, high triglycerides (TG), low high density lipoprotein cholesterol (HDL-c), hypertension (HTN), and diabetes mellitus (DM) affects overall CVD risk.

Our study takes a comprehensive approach by considering all these factors simultaneously, aiming to provide insights into their collective influence on cardiovascular health. This holistic analysis contributes to our understanding of how these diverse risk factors, when combined with smoking, interact to impact cardiovascular outcomes. Importantly, each of these individual risk factors has long been recognized for its independent contribution to CVD development [1, 8].

Building upon the existing body of knowledge, our study is designed to dissect the interplay between smoking and a spectrum of cardiovascular risk factors within the framework of both traditional and novel risk indicators. By employing a detailed analysis that encompasses serum cotinine levels for accurate smoking assessment [15, 16], alongside traditional risk factors and emerging indicators such as waist-to-height ratio (WHtR), visceral adiposity index (VAI), body shape index (ABSI), Conicity index (CI) and the triglyceride-glucose (TyG) index, we aim to show how smoking affects CVD risk in a number of different ways. This comprehensive approach allows us to examine the synergistic effects of smoking and various cardiovascular risk factors, offering a subtle perspective on their collective contribution to cardiovascular health.

Through leveraging the dataset provided by the National Health and Nutrition Examination Survey (NHANES), our investigation seeks to provide a contemporary analysis that reflects the current dynamics of cardiovascular risk factors and smoking patterns. By integrating a wide array of risk determinants and utilizing biomarker data, we aspire to refine our understanding of the complex relationship between smoking, cardiovascular risk factors, and CVD risk. This study aims not only to bridge existing research gaps but also to contribute valuable insights that could inform more targeted and effective CVD prevention and management strategies. Thus, our study positions itself at the intersection of traditional epidemiological research and the evolving landscape of cardiovascular risk assessment, aiming to contribute to the broader effort of mitigating the global burden of CVD.

## Methods

### Study design

Cross-sectional study.

### Data source

The NHANES, conducted by the Centers for Disease Control and Prevention (CDC), is a valuable tool for understanding the health and nutrition of the United States of America (USA) population. Our study utilized NHANES data, which is collected every two years to assess nutrition and health in the USA. The National Centre for Health Statistics (NCHS) is in charge of running NHANES, and it employs a sophisticated method to choose participants who accurately reflect the population. The NCHS Research Ethics Review Board approved NHANES study protocols, and all participants or their parents or guardians (if under 16) provided consent. Detailed NHANES study designs and data are accessible at www.cdc.gov/nchs/nhanes/index.htm (accessed on July 10, 2023).

### Study population

In this study, we utilized cross-sectional data gathered from multiple survey cycles spanning 2011 to 2018 as part of the NHANES. Initially, a total of 39,156 individuals were included in these survey cycles. After examining serum cotinine levels, 8,982 individuals had no serum cotinine and were subsequently excluded. Smoking status was ascertained based on serum cotinine concentration, whereby a concentration of ≥ 10ng/mL indicated smoker, while a concentration of < 10ng/mL indicated nonsmoker [15, 16].

**Fig. 1:**
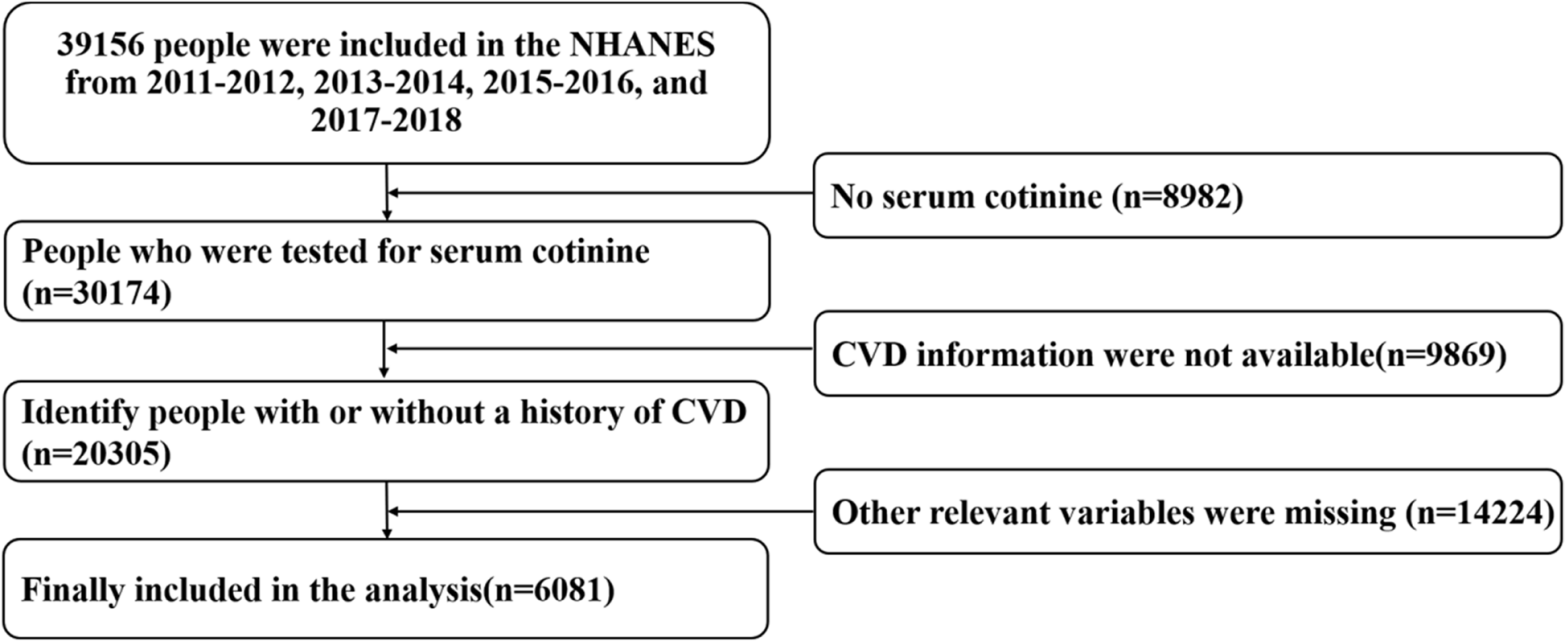
Illustrating the recruitment flow

Among the remaining 30,174 participants, 9,869 were excluded due to the unavailability of cardiovascular information, further narrowing down the sample to 20,305 participants. After removing 14,224 individuals with missing relevant variables, this stringent selection process ultimately led to the enrollment of 6,081 participants for the study, as depicted in Fig. 1.

Out of the 6,081 participants who were included in the study, 5,427 individuals were found to be free of CVD, whereas 654 participants had CVD. The NHANES questionnaire dataset was instrumental in ascertaining the presence of conditions such as coronary heart disease, angina pectoris, and heart attack. The assessment involved questions (MCQ160 b-e) that inquired whether a healthcare professional had ever diagnosed the participant with congestive heart failure, coronary heart disease, angina, or a heart attack.

Individuals who indicated the absence of these conditions were categorized as not having CVD. Conversely, if a participant reported one or more of these conditions, they were classified as having CVD.

### Data collection

NHANES collected data covering demographic information, clinical assessments, and laboratory biomarkers. This encompassed details on smoking habits, obesity, blood pressure, serum cotinine levels, lipid profiles, blood glucose levels, liver and kidney function, hematological parameters, alcohol consumption, hypertension, diabetes, chronic kidney disease (CKD), and medication usage.

Hypertension was identified by either self-reported history (BPQ020: ‘Have you ever been told by a doctor or other health professional that you had hypertension?’ answered yes), current use of blood pressure medications, or having a systolic blood pressure ≥ 140mmHg or diastolic blood pressure ≥ 90mmHg.

Diabetes was recognized through self-reported history (DIQ010: ‘Have you ever been told by a doctor or other health professional that you had diabetes other than during pregnancy?’ answered yes), use of hypoglycemic drugs or insulin, or a fasting blood glucose level ≥ 7 mmol/L.

Chronic kidney disease was determined by either a self-reported history of kidney disease (KIQ022: ‘Have you ever been told by a doctor or other health professional that you had a weak or failing kidney, do not include kidney stones, bladder infections, or incontinence?’ answered yes) or an estimated glomerular filtration rate (eGFR) < 60 ml/ (min*1.73m^2^), calculated using the 2021 CKD-EPI Creatinine equation [17].

### Data analysis

Statistical analyses were performed using R version 4.2.0 and EmpowerStats software, with a significance level set at *p* < 0.05. Continuous variables were summarized as mean ± SD for those with a normal distribution and median (IQR) for variables with a non-normal distribution. Categorical variables were expressed as percentages (%). The chi-squared test was used to compare baseline characteristics between CVD and non-CVD groups.

To explore the relationship between serum cotinine levels and CVD risk, we employed multivariable logistic regression models, adjusting for factors known to influence CVD. During initial analysis, we noted an unusually large odds ratio of 103.97 for WHtR in the non-adjusted model. A thorough review of the dataset revealed no significant outliers in WHtR measurements that could account for this high ratio. Consequently, we adjusted the WHtR’s dimension conversion ratio to 10, which refined its scale and impact within the model. This methodological adjustment led to a more interpretable revised odds ratio of 1.59 for WHtR in the non-adjusted model, substantially reducing the initial estimate and improving the accuracy of our findings. This adjustment is detailed in Table 6 in the results section.

Relationships were visualized using a generalized additive model with smooth curve fitting. A two-step conjoint analysis was conducted to examine the combined effects of smoking with multiple cardiovascular risk indicators and CVD risk factors on CVD risk.

The metric performance of various CVD risk indicators, including waist-to-height ratio (WHtR), Conicity index (CI), visceral adiposity index (VAI), body shape index (ABSI), triglyceride glucose index (TyG-Index), neutrophil-to-lymphocyte ratio (NLR), and neutrophil, was evaluated. This evaluation was conducted using receiver operating characteristic (ROC) curves.

## Results

### Characteristics of the study population

**Fig. 2:**
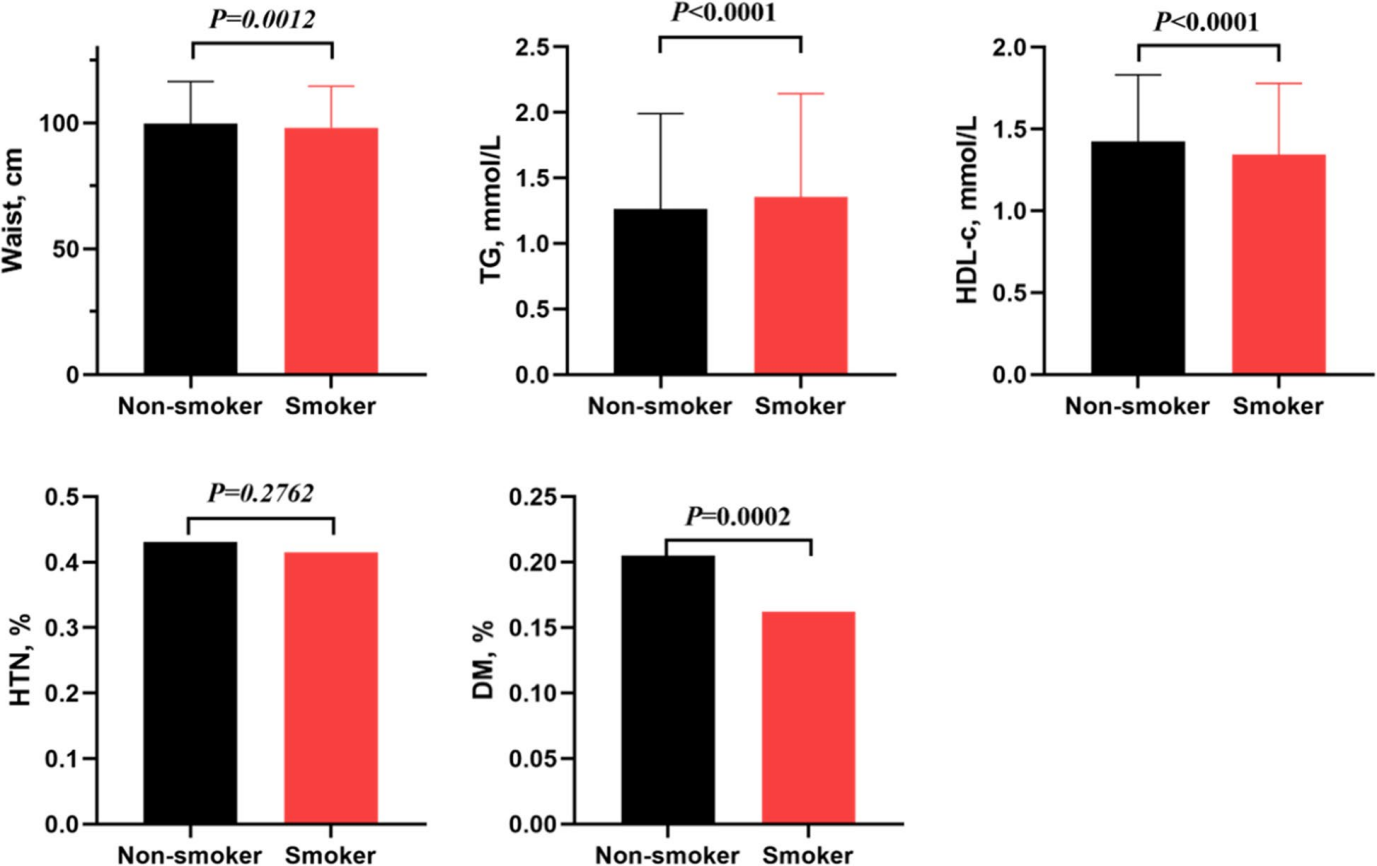
The distribution of various cardiovascular risk factors in smoker and non-smoker groups

Table 1 provides an overview of the study population’s characteristics, emphasizing differences between the CVD and non-CVD groups. The participant age span was notably broad, ranging from the youngest at 20 years to the eldest at 80 years, underscoring the inclusivity of our analysis and allowing for a comprehensive exploration of cardiovascular risk factors across a diverse spectrum of adult life stages. The CVD group, on average, was older and had a higher proportion of males. Non-Hispanic white individuals were more prevalent in the CVD group. Several health indicators, including SBP, BMI, and waist circumference, were notably higher in the CVD group. Alcohol consumption patterns varied, with more CVD participants reporting no alcohol use. Conditions such as HTN, DM, and CKD were strongly associated with CVD, exhibiting higher prevalence rates in the CVD group. Lipid profiles (TG, HDL-c, LDL-c, and TC), as well as other biomarkers (HbA1c, FPG, albumin, BUN, creatinine, uric acid, WBC, neutrophils, hemoglobin, and platelets), showed significant differences between the two groups. Medication usage, including anti-platelet drugs, β-blockers, statins, diuretics, and anticoagulants, was also significantly associated with CVD. Refer to Fig. 2, for a visual representation of various cardiovascular risk factors in both smoker and non-smoker groups.

**Table 1:** Characteristics of the study population

	Non-CVD	CVD	*P*-value
Participant	5427	654	
Age, years	47.52 ± 17.06	65.91 ± 12.39	< 0.001
Sex			0.002
Male	2656 (48.94%)	363 (55.50%)	
Female	2771 (51.06%)	291 (44.50%)	
Race			< 0.001
Mexican American	748 (13.78%)	59 (9.02%)	
Other Hispanic	628 (11.57%)	72 (11.01%)	
Non-Hispanic White	2118 (39.03%)	339 (51.83%)	
Non-Hispanic Black	1105 (20.36%)	141 (21.56%)	
Other Race	828 (15.26%)	43 (6.57%)	
SBP, mmHg	122.78 ± 17.50	130.87 ± 21.09	< 0.001
DBP, mmHg	69.49 ± 12.07	65.43 ± 15.53	< 0.001
BMI, kg/m^2^	28.91 ± 6.86	30.57 ± 7.25	< 0.001
Waist, cm	98.63 ± 16.46	106.27 ± 16.14	< 0.001
Alcohol status			< 0.001
No	1499 (27.62%)	226 (34.56%)	
Yes	3928 (72.38%)	428 (65.44%)	
HTN			< 0.001
No	3335 (61.45%)	149 (22.78%)	
Yes	2092 (38.55%)	505 (77.22%)	
DM			< 0.001
No	4520 (83.29%)	376 (57.49%)	
Yes	907 (16.71%)	278 (42.51%)	
CKD			< 0.001
No	5082 (93.64%)	458 (70.03%)	
Yes	345 (6.36%)	196 (29.97%)	
HDL-c, mmol/L	1.41 ± 0.42	1.33 ± 0.42	< 0.001
TG, mmol/L	1.27 ± 0.74	1.41 ± 0.77	< 0.001
LDL-c, mmol/L	2.95 ± 0.90	2.57 ± 0.94	< 0.001
TC, mmol/L	4.95 ± 1.03	4.55 ± 1.09	< 0.001
COT, ng/mL	0.03 (0.01–4.14)	0.04 (0.01–80.68)	0.003
HbA1c, %	5.73 ± 1.08	6.30 ± 1.39	< 0.001
FPG, mmol/L	5.96 ± 1.83	6.87 ± 2.62	< 0.001
A, g/L	42.78 ± 3.28	41.51 ± 3.32	< 0.001
ALT, U/L	21.00 (16.00–28.00)	20.00 (16.00–26.00)	0.111
AST, U/L	22.00 (19.00–27.00)	23.00 (19.00–28.00)	0.101
BUN, mmol/L	4.69 ± 1.84	6.25 ± 3.02	< 0.001
CR, mmol/L	72.49 (61.00-85.75)	87.52 (70.72–105.20)	< 0.001
TB, umol/L	11.38 ± 4.99	11.46 ± 5.76	0.702
UA, umol/L	322.18 ± 82.47	357.13 ± 92.93	< 0.001
WBC, *10^9^/L	6.76 ± 2.16	7.48 ± 4.97	< 0.001
Lym, *10^9^/L	1.90 (1.60–2.40)	1.80 (1.40–2.40)	0.357
Neut, *10^9^/L	3.92 ± 1.60	4.46 ± 1.70	< 0.001
Hb, g/dL	14.11 ± 1.50	13.81 ± 1.64	< 0.001
PLT, *10^9^/L	235.81 ± 60.30	218.94 ± 64.77	< 0.001
MPV, fL	8.42 ± 0.93	8.53 ± 0.94	0.004
Anti-platelet			< 0.001
No	5374 (99.02%)	523 (79.97%)	
Yes	53 (0.98%)	131 (20.03%)	
β-blockers			0.003
No	5379 (99.12%)	640 (97.86%)	
Yes	48 (0.88%)	14 (2.14%)	
ACEI/ARB			0.888
No	5004 (92.21%)	602 (92.05%)	
Yes	423 (7.79%)	52 (7.95%)	
Statin			< 0.001
No	4635 (85.41%)	383 (58.56%)	
Yes	792 (14.59%)	271 (41.44%)	
Diuretic			0.002
No	5086 (93.72%)	592 (90.52%)	
Yes	341 (6.28%)	62 (9.48%)	
Anticoagulant			< 0.001
No	5403 (99.56%)	628 (96.02%)	
Yes	24 (0.44%)	26 (3.98%)	

*SBP* Systolic blood pressure, *DBP* Diastolic blood pressure, *BMI* Body mass index, *HTN* Hypertension, *DM* Diabetes mellitus, *CKD* Chronic kidney disease, *HDL-c* High density lipoprotein cholesterol, *TC* Total cholesterol, *LDL-c* Low density lipoprotein cholesterol, *COT*-Cotinine, *FPG* Fasting plasma glucose, *A* Albumin, *ALT* Alanine Aminotransferase, *AST* Aspartate Aminotransferase, *BUN* Blood Urea Nitrogen, *CR* Creatinine, *TB* Total bilirubin, *WBC* White blood cells, *Hb* Hemoglobin, *HbA1c*-Hemoglobin A1c, *PLT* platelets, *MPV* Mean platelets volume, *UA* Uric acid, *ACEI* Angiotensin converting enzyme inhibitor, *ARB* Angiotensin receptor blocker

### Exploring the associations between serum cotinine levels and CVD risk

**Fig. 3:**
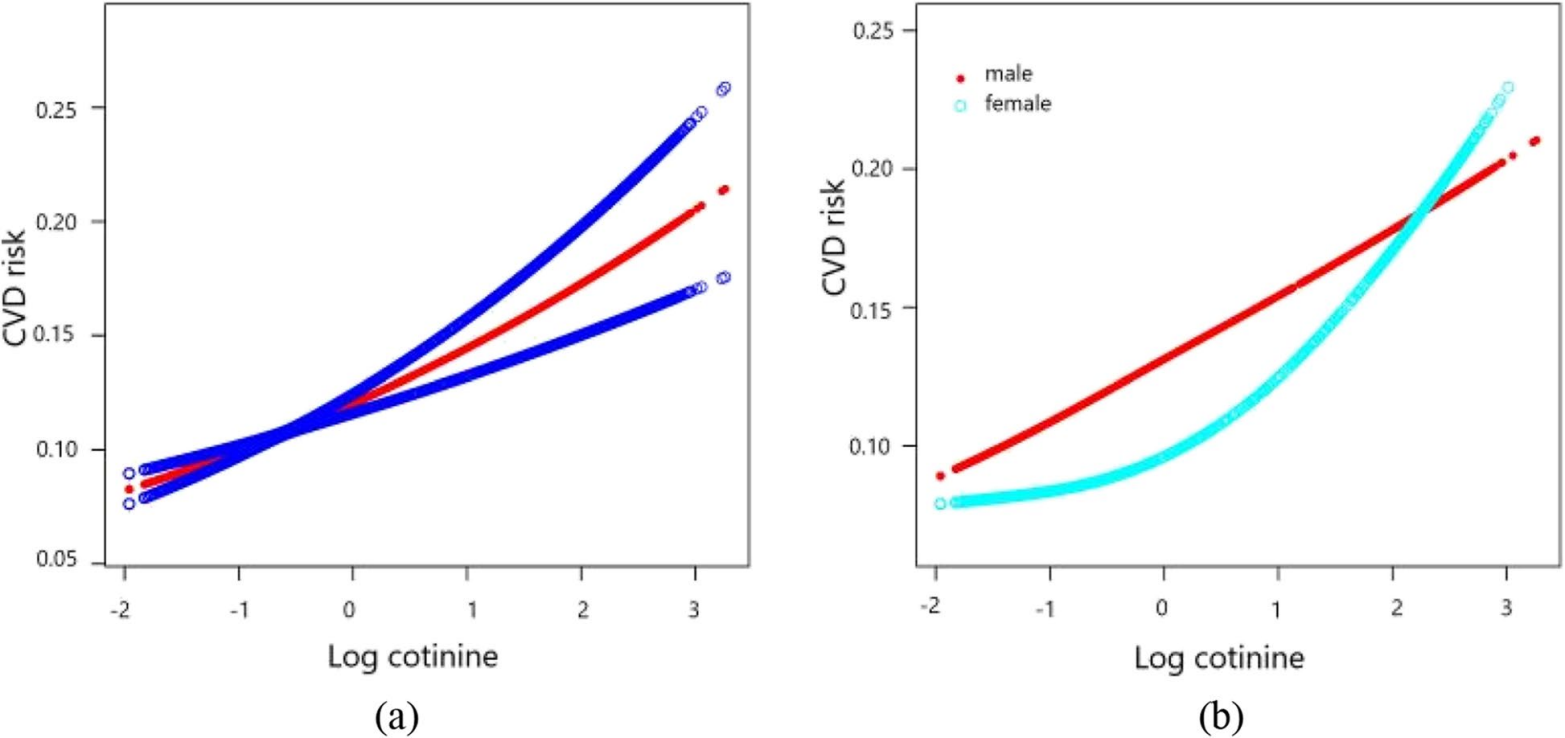
Visualizing the relationship between serum cotinine and cardiovascular disease risk

Table 2 and Fig. 3 present the association between serum cotinine levels and CVD risk through three logistic regression models. In the non-adjusted model, each unit rise in cotinine concentration corresponded to a 5% increase in CVD risk (OR = 1.05, *p* = 0.0273). After adjusting for covariates in the Adjust I Model, this risk substantially increased to 21% per unit rise (OR = 1.21, *p* < 0.0001). The full Adjust II Model, which took into account more variables, showed that the risk of CVD rose by 25% for every unit increase in cotinine levels (OR = 1.25, *p* < 0.0001). Serum cotinine quartiles showed that the highest quartile (Q4) was associated with a 2.33-fold increased risk of CVD compared to the reference (OR = 2.33, *p* < 0.0001), even after adjusting for confounding factors. Supplementary Material 1 provides a sex-stratified analysis to explore potential gender-based differences in the associations between serum cotinine levels and the risk of CVDs.

**Table 2:** Exploring the associations between serum cotinine levels and cardiovascular disease risk

Exposure	Non-adjusted OR (95%CI) *P*-value	Adjust I OR (95%CI) *P*-value	Adjust II OR (95%CI) *P*-value
Log Cotinine	1.05 (1.01, 1.10) 0.0273	1.21 (1.14, 1.27) < 0.0001	1.25 (1.18, 1.33) < 0.0001
Log Cotinine (quartile)			
Q1	Ref	Ref	Ref
Q2	0.81 (0.63, 1.04) 0.0979	0.97 (0.74, 1.28) 0.8393	0.94 (0.69, 1.27) 0.6733
Q3	0.89 (0.71, 1.10) 0.2854	1.25 (0.99, 1.60) 0.0654	1.16 (0.89, 1.52) 0.2683
Q4	1.06 (0.86, 1.31) 0.5698	2.07 (1.63, 2.64) < 0.0001	2.33 (1.77, 3.07) < 0.0001
*P* for trend	0.6010	< 0.0001	< 0.0001

Non-adjusted model adjust for: None

Adjust I model adjust for: Sex; Age; Race

Adjust II model adjust for: Sex; Age; Race; SBP; DBP; BMI; Waist; HDL-c; TG; LDL-c; TC; HbA1c; FPG; A; ALT; AST; BUN; CR; TB; UA; Alcohol; HTN; DM; CKD; Anti-platelet; β-blockers; ACEI/ARB; Statin; Diuretic; Anticoagulant

### Impact of smoking with multiple CVD risk factors on CVD risk

Table 3 emphasizes significant associations between common cardiovascular risk factors and CVD risk. Notably, after carefully considering various factors, in the fully adjusted Model II, we observed significant associations between waist measurements, TG, HDL-c, HTN, and DM and an increased risk of CVD. Specifically, waist circumference exhibited a notable effect, with each one-unit increase corresponding to a 1.01-fold higher risk of CVD (*p* = 0.0027). TG levels were also found to be influential, where a 1.17-fold increase in risk was associated with each unit increase (*p* = 0.0233). HDL-c showed a significant inverse relationship, with each unit increase corresponding to a 0.59-fold decrease in risk (*p* < 0.0001). Furthermore, the presence of HTN was strongly associated with CVD, with individuals with HTN exhibiting a 1.57-fold increased CVD risk compared to those without HTN (*p* = 0.0003). Similarly, DM had a significant impact, with individuals with DM showing a 1.40-fold higher CVD risk compared to those without DM (*p* = 0.0017).

**Table 3:** Relationship between common cardiovascular risk factors and cardiovascular disease risk

Exposure	Non-adjusted OR (95%CI) *P*-value	Adjust I OR (95%CI) *P*-value	Adjust II OR (95%CI) *P*-value
Waist	1.03 (1.02, 1.03) < 0.0001	1.02 (1.02, 1.03) < 0.0001	1.01 (1.00, 1.02) 0.0027
Waist (quartile)			
Q1	Ref	Ref	Ref
Q2	1.86 (1.39, 2.49) < 0.0001	1.13 (0.83, 1.55) 0.4273	0.94 (0.67, 1.32) 0.7207
Q3	2.55 (1.93, 3.37) < 0.0001	1.30 (0.96, 1.76) 0.0886	0.99 (0.71, 1.39) 0.9700
Q4	3.93 (3.01, 5.13) < 0.0001	2.22 (1.66, 2.96) < 0.0001	1.42 (1.02, 1.98) 0.0404
P for trend	< 0.0001	< 0.0001	0.0064
TG	1.26 (1.14, 1.39) < 0.0001	1.29 (1.15, 1.45) < 0.0001	1.17 (1.02, 1.34) 0.0233
TG (quartile)			
Q1	Ref	Ref	Ref
Q2	1.44 (1.12, 1.85) 0.0043	1.17 (0.89, 1.53) 0.2637	0.96 (0.71, 1.29) 0.7766
Q3	1.64 (1.28, 2.10) < 0.0001	1.32 (1.01, 1.72) 0.0458	1.02 (0.76, 1.38) 0.8958
Q4	1.83 (1.43, 2.32) < 0.0001	1.59 (1.22, 2.07) 0.0006	1.17 (0.86, 1.59) 0.3145
P for trend	< 0.0001	0.0003	0.2230
HDL-c	0.58 (0.47, 0.72) < 0.0001	0.40 (0.32, 0.52) < 0.0001	0.59 (0.45, 0.76) < 0.0001
HDL-c (quartile)			
Q1	Ref	Ref	Ref
Q2	0.73 (0.59, 0.91) 0.0040	0.66 (0.52, 0.84) 0.0006	0.73 (0.56, 0.95) 0.0184
Q3	0.53 (0.42, 0.66) < 0.0001	0.43 (0.33, 0.56) < 0.0001	0.56 (0.42, 0.75) 0.0001
Q4	0.56 (0.45, 0.70) < 0.0001	0.37 (0.28, 0.48) < 0.0001	0.54 (0.40, 0.72) < 0.0001
P for trend	< 0.0001	< 0.0001	< 0.0001
HTN			
No	Ref	Ref	Ref
Yes	5.40 (4.46, 6.54) < 0.0001	2.41 (1.95, 2.96) < 0.0001	1.57 (1.23, 1.99) 0.0003
DM			
No	Ref	Ref	Ref
Yes	3.68 (3.11, 4.37) < 0.0001	2.14 (1.78, 2.58) < 0.0001	1.40 (1.14, 1.74) 0.0017

Non-adjusted model adjust for: None

Adjust I model adjust for: Sex; Age; Race

Adjust II model adjust for: Sex; Age; Race; A; ALT; AST; BUN; CR; TB; UA; Alcohol; Smoke; Anti-platelet; β-blockers; ACEI/ARB; Statin; Diuretic; Anticoagulant

Table 4 explores the combined effects of smoking with other cardiovascular risk factors. In the fully adjusted Model II, it reveals significant increases in the risk of CVD for individuals who smoke in conjunction with central obesity, high TG levels, and lower HDL-c levels. Specifically, smoking combined with central obesity showed a substantial 3.04-fold increase in the risk of CVD (*p* < 0.0001). Smokers with higher TG levels experienced a significant 2.72-fold increase in CVD risk (*p* < 0.0001), while those with lower HDL-c levels saw a striking 3.82-fold increase in CVD risk (*p* < 0.0001). Hypertension and diabetes significantly elevated CVD risk in both non-smokers and smokers, remaining significant after adjustments (HTN: non-smokers had a 1.61-fold increase, *p* = 0.0008, while smokers showed a 3.55-fold increase, *p* < 0.0001; DM: non-smokers exhibited a 1.48-fold increase, *p* = 0.0012, while smokers had a 2.93-fold increase, *p* < 0.0001).

**Table 4:** Effects of smoking combined with common cardiovascular risk factors, grouped based on clinical cut-off points, on CVD risk

Exposure	Non-adjusted OR (95%CI) *P*-value	Adjust I OR (95%CI) *P*-value	Adjust II OR (95%CI) *P*-value
Non-smoker, non-central obesity	Ref	Ref	Ref
Non-smoker, central obesity	3.70 (2.51, 5.46) < 0.0001	1.86 (1.22, 2.81) 0.0036	1.23 (0.78, 1.93) 0.3747
Smoker, non-central obesity	1.23 (0.65, 2.32) 0.5336	1.47 (0.75, 2.90) 0.2611	1.39 (0.67, 2.87) 0.3796
Smoker, central obesity	4.86 (3.22, 7.33) < 0.0001	3.93 (2.53, 6.12) < 0.0001	3.04 (1.87, 4.94) < 0.0001
Non-smoker, lower TG	Ref	Ref	Ref
Non-smoker, higher TG	1.33 (1.07, 1.66) 0.0104	1.35 (1.06, 1.71) 0.0140	1.29 (0.99, 1.68) 0.0631
Smoker, lower TG	1.19 (0.96, 1.48) 0.1162	1.90 (1.49, 2.43) < 0.0001	2.31 (1.75, 3.04) < 0.0001
Smoker, higher TG	1.64 (1.21, 2.22) 0.0015	2.80 (2.00, 3.93) < 0.0001	2.72 (1.86, 3.98) < 0.0001
Non-smoker, higher HDL-c	Ref	Ref	Ref
Non-smoker, lower HDL-c	1.65 (1.27, 2.16) 0.0002	2.25 (1.67, 3.03) < 0.0001	1.69 (1.21, 2.35) 0.0022
Smoker, higher HDL-c	1.22 (1.00, 1.49) 0.0543	1.97 (1.57, 2.48) < 0.0001	2.22 (1.72, 2.87) < 0.0001
Smoker, lower HDL-c	1.75 (1.21, 2.54) 0.0030	3.73 (2.47, 5.64) < 0.0001	3.82 (2.43, 6.01) < 0.0001
Non-smoker, non-HTN	Ref	Ref	Ref
Non-smoker, HTN	5.87 (4.67, 7.38) < 0.0001	2.38 (1.86, 3.05) < 0.0001	1.61 (1.22, 2.13) 0.0008
Smoker, non-HTN	1.54 (1.08, 2.18) 0.0163	1.95 (1.34, 2.82) 0.0004	2.42 (1.63, 3.59) < 0.0001
Smoker, HTN	6.91 (5.23, 9.12) < 0.0001	4.76 (3.53, 6.42) < 0.0001	3.55 (2.53, 4.98) < 0.0001
Non-smoker, non-DM	Ref	Ref	Ref
Non-smoker, DM	4.01 (3.29, 4.88) < 0.0001	2.36 (1.90, 2.92) < 0.0001	1.48 (1.17, 1.88) 0.0012
Smoker, non-DM	1.45 (1.16, 1.82) 0.0013	2.27 (1.76, 2.92) < 0.0001	2.49 (1.88, 3.30) < 0.0001
Smoker, DM	4.42 (3.21, 6.09) < 0.0001	3.95 (2.79, 5.60) < 0.0001	2.93 (1.99, 4.32) < 0.0001

Non-adjusted model adjust for: None

Adjust I model adjust for: Sex; Age; Race

Adjust II model adjust for: Sex; Age; Race; A; ALT; AST; BUN; CR; TB; UA; Alcohol; Anti-platelet; β-blockers; ACEI/ARB; Statin; Diuretic; Anticoagulant

Table 5 provides a comprehensive analysis of smoking combined with multiple cardiovascular risk factors in groups. Group q1, characterized by smoking, central obesity, elevated TG, lower HDL-c, and hypertension, exhibits the highest CVD risk, with an odds ratio of 14.18 in the Adjust II model. This risk pattern is consistent across most groups, emphasizing the association between smoking and multiple risk factors with increased CVD susceptibility. However, following Adjustment II, some groups, like m1 (smoking, central obesity, higher TG, DM), r1 (smoking, central obesity, higher TG, lower HDL-c, DM), and s1 (smoking, central obesity, higher TG, lower HDL-c, HTN, DM), lost statistical significance in their CVD risk. This intriguing finding highlights the complex interplay of risk factors in shaping CVD outcomes.

**Table 5:** Combined effects of smoking and multiple cardiovascular risk factors on cardiovascular disease risk

Exposure	Non-adjusted OR (95%CI) *P*-value	Adjust I OR (95%CI) *P*-value	Adjust II OR (95%CI) *P*-value
Group a0	Ref	Ref	Ref
Group a1	5.09 (3.14, 8.24) < 0.0001	4.32 (2.47, 7.56) < 0.0001	2.96 (1.41, 6.22) 0.0041
Group b0	Ref	Ref	Ref
Group b1	5.93 (3.49, 10.08) < 0.0001	6.17 (3.25, 11.69) < 0.0001	4.13 (1.72, 9.91) 0.0015
Group c0	Ref	Ref	Ref
Group c1	19.37 (10.04, 37.38) < 0.0001	8.16 (4.05, 16.47) < 0.0001	4.77 (2.10, 10.85) 0.0002
Group d0	Ref	Ref	Ref
Group d1	11.55 (6.90, 19.33) < 0.0001	4.96 (2.81, 8.74) < 0.0001	2.48 (1.11, 5.56) 0.0274
Group e0	Ref	Ref	Ref
Group e1	2.01 (1.24, 3.26) 0.0046	3.58 (2.10, 6.08) < 0.0001	3.20 (1.76, 5.81) 0.0001
Group f0	Ref	Ref	Ref
Group f1	7.58 (4.99, 11.50) < 0.0001	6.21 (3.90, 9.91) < 0.0001	3.86 (2.10, 7.07) < 0.0001
Group g0	Ref	Ref	Ref
Group g1	4.82 (2.93, 7.91) < 0.0001	5.59 (3.25, 9.60) < 0.0001	3.38 (1.78, 6.41) 0.0002
Group h0	Ref	Ref	Ref
Group h1	7.99 (4.82, 13.22) < 0.0001	7.61 (4.35, 13.32) < 0.0001	4.28 (2.11, 8.70) < 0.0001
Group i0	Ref	Ref	Ref
Group i1	4.14 (2.24, 7.65) < 0.0001	5.52 (2.84, 10.73) < 0.0001	3.81 (1.80, 8.07) 0.0005
Group j0	Ref	Ref	Ref
Group j1	16.41 (10.68, 25.20) < 0.0001	7.92 (4.89, 12.83) < 0.0001	3.73 (1.96, 7.10) < 0.0001
Group k0	Ref	Ref	Ref
Group k1	5.93 (3.20, 11.00) < 0.0001	5.69 (2.73, 11.86) < 0.0001	3.75 (1.29, 10.91) 0.0153
Group l0	Ref	Ref	Ref
Group l1	17.34 (8.43, 35.68) < 0.0001	7.37 (3.38, 16.08) < 0.0001	6.10 (1.82, 20.41) 0.0033
Group m0	Ref	Ref	Ref
Group m1	12.33 (6.47, 23.47) < 0.0001	6.72 (3.25, 13.89) < 0.0001	3.19 (0.96, 10.55) 0.0579
Group n0	Ref	Ref	Ref
Group n1	9.11 (5.02, 16.55) < 0.0001	8.08 (4.18, 15.59) < 0.0001	4.42 (1.88, 10.40) 0.0007
Group o0	Ref	Ref	Ref
Group o1	4.36 (2.03, 9.37) 0.0002	6.61 (2.92, 14.96) < 0.0001	3.42 (1.32, 8.83) 0.0110
Group p0	Ref	Ref	Ref
Group p1	12.09 (5.44, 26.85) < 0.0001	9.34 (3.97, 21.93) < 0.0001	4.65 (1.52, 14.22) 0.0071
Group q0	Ref	Ref	Ref
Group q1	22.15 (9.40, 52.19) < 0.0001	10.60 (4.15, 27.06) < 0.0001	14.18 (2.23, 90.05) 0.0049
Group r0	Ref	Ref	Ref
Group r1	10.64 (4.44, 25.47) < 0.0001	7.93 (2.84, 22.20) < 0.0001	2.33 (0.36, 15.15) 0.3762
Group s0	Ref	Ref	Ref
Group s1	23.17 (7.32, 73.39) < 0.0001	18.37 (4.30, 78.48) < 0.0001	5.29 (0.13, 215.64) 0.3784

Non-adjusted model adjust for: None

Adjust I model adjust for: Sex; Age; Race

Adjust II model adjust for: Sex; Age; Race; A; ALT; AST; BUN; CR; TB; UA; Alcohol; Anti-platelet; β-blockers; ACEI/ARB; Statin; Diuretic; Anticoagulant

(**a**): smoke, central obesity, higher TG; (**b**): smoke, central obesity, lower HDL-c; (**c**): smoke, central obesity, HTN; (**d**): smoke, central obesity, DM; (**e**): smoke, higher TG, lower HDL-c; (**f**): smoke, higher TG, HTN; (**g**): smoke, higher TG, DM; (**h**): smoke, lower HDL-c, HTN; (**i**): smoke, lower HDL-c, DM; (**j**): smoke, HTN, DM; (**k**): smoke, central obesity, higher TG, lower HDL-c; (**l**): smoke, central obesity, higher TG, HTN; (**m**): smoke, central obesity, higher TG, DM; (**n**): smoke, higher TG, lower HDL-c, HTN; (**o**): smoke, higher TG, lower HDL-c, DM; (**p**): smoke, lower HDL-c, HTN, DM; (**q**): smoke, central obesity, higher TG, lower HDL-c, HTN; (**r**): smoke, central obesity, higher TG, lower HDL-c, DM; (**s**): smoke, central obesity, higher TG, lower HDL-c, HTN,DM

Group 0 is negative and group 1 is positive

### Associations between emerging CVD risk indicators and CVD risk

Our analysis, conducted within the fully adjusted model (Adjust II), revealed compelling associations between various CVD risk indicators and the risk of CVD. Remarkably, each unit increase in WHtR was linked to a substantial 1.25-fold increase in CVD risk (*p* = 0.0001), underscoring its significant predictive value. Additionally, VAI displayed a robust association, with a 1.10-fold increase in CVD risk (*p* = 0.0024) for each unit rise in this indicator. ABSI also exhibited a noteworthy effect, with a 1.36-fold increase in CVD risk (*p* = 0.0099) per unit increase. Furthermore, CI, TyG-Index, neutrophils, NLR, and MPV all demonstrated significant associations with CVD risk (*P* < 0.05), underscoring their clinical relevance. However, white blood cell count and PLR, while initially indicated increased CVD risk, lost their significance after adjustments. Lymphocytes were not associated with an increased risk of CVD in all models, making them not a suitable biomarker for CVD risk assessment (Table 6).

**Table 6:** Assessment of cardiovascular disease risk using various cardiovascular risk indicators

Exposure	Non-adjusted OR (95%CI) *P*-value	Adjust I OR (95%CI) *P*-value	Adjust II OR (95%CI) *P*-value
WHtR10	1.59 (1.47, 1.72) < 0.0001	1.48(1.35, 1.63) < 0.0001	1.25 (1.11, 1.40) 0.0001
VAI	1.15 (1.10, 1.21) < 0.0001	1.18 (1.12, 1.25) < 0.0001	1.10 (1.03, 1.17) 0.0024
ABSI	4.81 (4.01, 5.76) < 0.0001	1.72 (1.39, 2.13) < 0.0001	1.36 (1.08, 1.73) 0.0099
CI	4.51 (3.81, 5.35) < 0.0001	2.20 (1.81, 2.68) < 0.0001	1.53 (1.22, 1.92) 0.0002
TyG	1.75 (1.55, 1.98) < 0.0001	1.53 (1.33, 1.77) < 0.0001	1.26 (1.07, 1.49) 0.0053
WBC	1.10 (1.06, 1.13) < 0.0001	1.12 (1.08, 1.17) < 0.0001	1.03 (1.00, 1.07) 0.0882
LYM	1.01 (0.98, 1.05) 0.3867	1.02 (0.98, 1.05) 0.3727	1.01 (0.98, 1.04) 0.6601
NEUT	1.19 (1.14, 1.25) < 0.0001	1.21 (1.15, 1.28) < 0.0001	1.09 (1.03, 1.16) 0.0028
PLR	1.00 (1.00, 1.00) 0.0444	1.00 (1.00, 1.00) 0.5565	1.00 (1.00, 1.00) 0.9849
NLR	1.38 (1.30, 1.46) < 0.0001	1.18 (1.10, 1.25) < 0.0001	1.08 (1.01, 1.16) 0.0344
MPV	1.13 (1.04, 1.23) 0.0044	1.16 (1.06, 1.27) 0.0018	1.14 (1.03, 1.26) 0.0139

Non-adjusted model adjust for: None

Adjust I model adjust for: Sex; Age; Race

Adjust II model adjust for: Sex; Age; Race; A; ALT; AST; BUN; CR; TB; UA; Alcohol; Smoke; Anti-platelet; β-blockers; ACEI/ARB; Statin; Diuretic; Anticoagulant

### The combined effects of smoking and CVD risk indicators on CVD risk assessment

In the fully adjusted Model II, our analysis revealed significant associations between various cardiovascular risk indicators and the risk of CVD, particularly when considering the combined effects of smoking with these factors. Individuals with a high WHtR, both non-smokers and smokers, exhibited an increased CVD risk, with smokers facing a substantial 2.51-fold increase in risk (*p* < 0.0001). High VAI also played a significant role, with smokers showing a remarkable 3.05-fold increase in CVD risk (*p* < 0.0001).

Moreover, in the context of ABSI and the CI, both non-smokers and smokers exhibited increased CVD risk, but the risk was notably higher among smokers. Smokers with a high ABSI had a 2.98-fold increase in CVD risk (*p* < 0.0001), and those with a high CI had a 3.28-fold increase in CVD risk (*p* < 0.0001).

Additionally, considering the TyG-Index, Neutrophil, NLR, and MPV, both non-smokers and smokers exhibited increased CVD risk. Still, the risk was significantly higher among smokers. Smokers with high TyG-Index had a 2.40-fold increase in CVD risk (*p* < 0.0001), while those with high Neutrophil and NLR had a 3.13 and 2.75-fold increase in CVD risk, respectively (*p* < 0.0001 for both). Smoking with high MPV was associated with a 2.47-fold increase in CVD risk (*p* < 0.0001) (Table 7).

**Table 7:** The combined effects of smoking and cardiovascular risk indicators on cardiovascular disease risk assessment

Exposure	Non-adjusted OR (95%CI) *P*-value	Adjust I OR (95%CI) *P*-value	Adjust II OR (95%CI) *P*-value
Non-smoker, low WHTR	Ref	Ref	Ref
Non-smoker, high WHTR	2.49 (2.04, 3.05) < 0.0001	1.72 (1.38, 2.14) < 0.0001	1.22 (0.95, 1.56) 0.1215
Smoker, low WHTR	1.67 (1.27, 2.18) 0.0002	2.37 (1.76, 3.19) < 0.0001	2.64 (1.90, 3.67) < 0.0001
Smoker, high WHTR	2.72 (2.05, 3.60) < 0.0001	3.20 (2.35, 4.35) < 0.0001	2.51 (1.77, 3.58) < 0.0001
Non-smoker, low VAI	Ref	Ref	Ref
Non-smoker, high VAI	1.61 (1.33, 1.95) < 0.0001	1.58 (1.28, 1.95) < 0.0001	1.34 (1.06, 1.70) 0.0158
Smoker, low VAI	1.19 (0.93, 1.54) 0.1675	1.87 (1.41, 2.48) < 0.0001	2.20 (1.61, 3.01) < 0.0001
Smoker, high VAI	1.92 (1.48, 2.49) < 0.0001	3.15 (2.35, 4.21) < 0.0001	3.05 (2.20, 4.22) < 0.0001
Non-smoker, low ABSI	Ref	Ref	Ref
Non-smoker, high ABSI	3.76 (3.01, 4.69) < 0.0001	1.50 (1.17, 1.92) 0.0012	1.33 (1.02, 1.74) 0.0324
Smoker, low ABSI	1.11 (0.76, 1.62) 0.5843	1.74 (1.16, 2.61) 0.0069	2.19 (1.43, 3.36) 0.0003
Smoker, high ABSI	4.69 (3.60, 6.11) < 0.0001	2.94 (2.21, 3.92) < 0.0001	2.98 (2.17, 4.10) < 0.0001
Non-smoker, low CI	Ref	Ref	Ref
Non-smoker, high CI	4.25 (3.38, 5.35) < 0.0001	1.88 (1.47, 2.41) < 0.0001	1.44 (1.10, 1.89) 0.0087
Smoker, low CI	1.27 (0.87, 1.85) 0.2084	1.81 (1.22, 2.69) 0.0035	2.25 (1.48, 3.44) 0.0002
Smoker, high CI	5.41 (4.11, 7.13) < 0.0001	3.78 (2.81, 5.09) < 0.0001	3.28 (2.36, 4.57) < 0.0001
Non-smoker, low TyG	Ref	Ref	Ref
Non-smoker, high TyG	1.61 (1.33, 1.95) < 0.0001	1.31 (1.06, 1.61) 0.0115	1.04 (0.82, 1.31) 0.7714
Smoker, low TyG	1.17 (0.88, 1.56) 0.2879	1.91 (1.38, 2.63) < 0.0001	2.21 (1.55, 3.17) < 0.0001
Smoker, high TyG	1.94 (1.51, 2.48) < 0.0001	2.57 (1.95, 3.38) < 0.0001	2.40 (1.76, 3.26) < 0.0001
Non-smoker, low NEUT	Ref	Ref	Ref
Non-smoker, high NEUT	1.88 (1.55, 2.28) < 0.0001	1.73 (1.40, 2.13) < 0.0001	1.44 (1.15, 1.81) 0.0016
Smoker, low NEUT	1.04 (0.76, 1.42) 0.8275	1.65 (1.17, 2.32) 0.0043	2.05 (1.41, 2.96) 0.0001
Smoker, high NEUT	2.09 (1.66, 2.64) < 0.0001	3.16 (2.44, 4.10) < 0.0001	3.13 (2.34, 4.20) < 0.0001
Non-smoker, low NLR	Ref	Ref	Ref
Non-smoker, high NLR	2.21 (1.83, 2.68) < 0.0001	1.58 (1.28, 1.95) < 0.0001	1.46 (1.16, 1.84) 0.0011
Smoker, low NLR	1.42 (1.10, 1.84) 0.0079	2.21 (1.66, 2.95) < 0.0001	2.70 (1.97, 3.69) < 0.0001
Smoker, high NLR	2.26 (1.74, 2.95) < 0.0001	2.72 (2.03, 3.65) < 0.0001	2.75 (1.97, 3.83) < 0.0001
Non-smoker, low MPV	Ref	Ref	Ref
Non-smoker, high MPV	1.31 (1.08, 1.58) 0.0057	1.32 (1.07, 1.62) 0.0082	1.27 (1.02, 1.59) 0.0348
Smoker, low MPV	1.35 (1.05, 1.72) 0.0194	2.24 (1.70, 2.96) < 0.0001	2.64 (1.94, 3.60) < 0.0001
Smoker, high MPV	1.44 (1.10, 1.89) 0.0089	2.30 (1.70, 3.11) < 0.0001	2.47 (1.77, 3.45) < 0.0001

Non-adjusted model adjust for: None

Adjust I model adjust for: Sex; Age; Race

Adjust II model adjust for: Sex; Age; Race; A; ALT; AST; BUN; CR; TB; UA; Alcohol; Anti-platelet; β-blockers; ACEI/ARB; Statin; Diuretic; Anticoagulant

### CVD detection metric performance of various CVD risk indicators

**Fig. 4:**
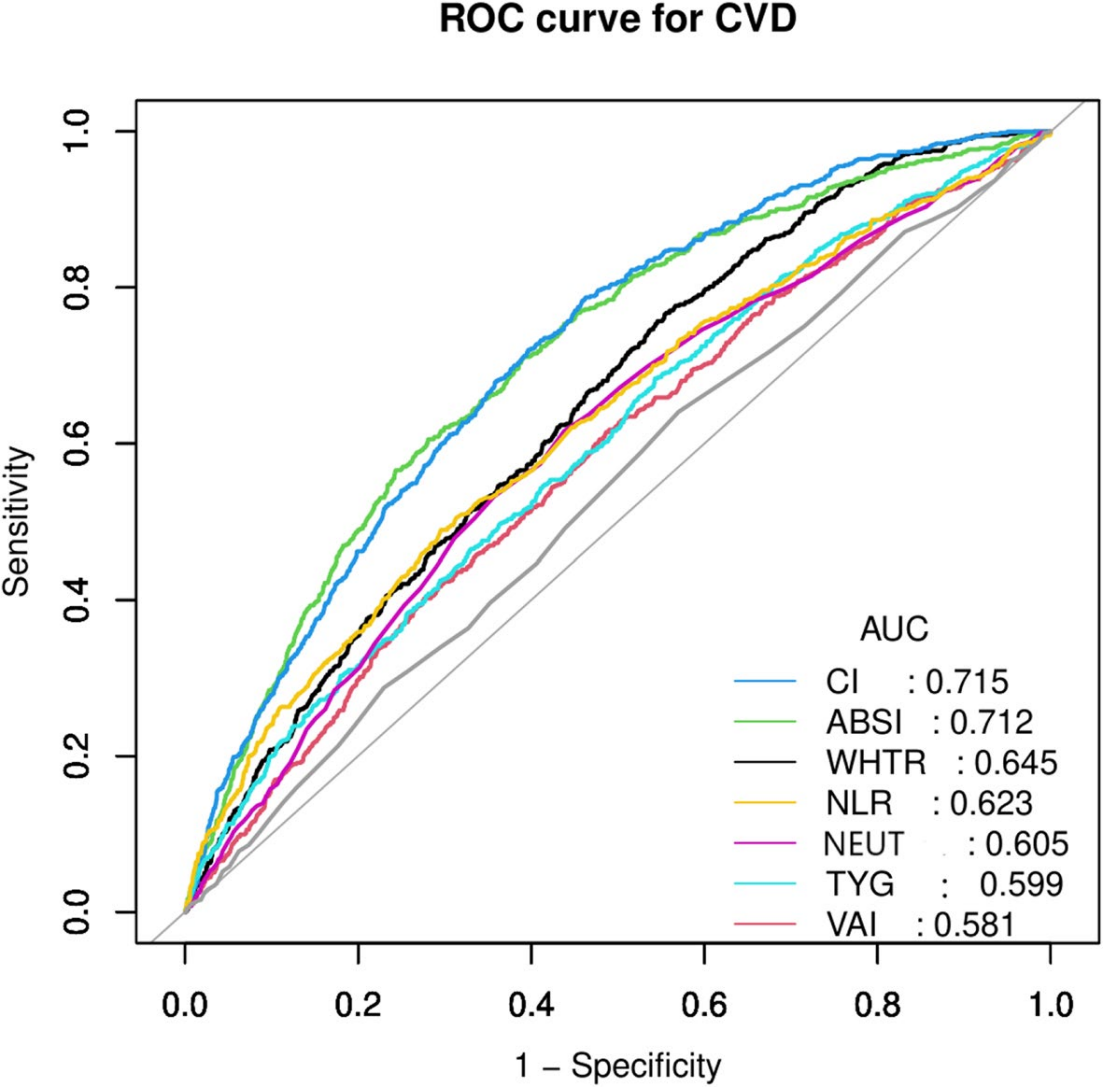
Receiver operating characteristics of various cardiovascular risk indicators

In our study, we conducted a thorough assessment of emerging CVD risk indicators presented in Table 6 for their effectiveness in detecting CVD using receiver operating characteristics (ROC). These indicators demonstrated a range of performance, with Area Under the Curve (AUC) values spanning from 0.5368 to 0.7118, indicating a moderate discriminatory ability (Fig. 4 and supplementary material 2).

## Discussion

In this cross-sectional study examining CVD risk factors, we investigated the association between diverse cardiovascular risk factors and indicators, and their synergistic impact with smoking on CVD risk. Our study involved a large sample of participants, and we employed multiple statistical models to explore these relationships comprehensively.

In our investigation into the association between serum cotinine levels and CVD risk, we found a significant dose-response relationship, with a notable 2.33-fold increase in CVD risk observed in the highest quartile of serum cotinine compared to the control group. This finding echoes the results of Omayma Alshaarawy’s study, which examined the relationship between secondhand smoke exposure, as measured by serum cotinine, and hypertension among non-smokers. Alshaarawy et al. found that higher serum cotinine levels were positively associated with hypertension in non-smokers, independently of various confounders [18]. Moreover, our study aligns with Affan Irfan’s and Ryan Brunetti’s research, which investigated specific cardiovascular outcomes associated with tobacco exposure, such as left atrial abnormalities and silent myocardial infarction, respectively [19, 20].

While these studies collectively shed light on the cardiovascular risks linked to smoking exposure, our investigation distinctively contributes by focusing specifically on the dose-response relationship between serum cotinine levels and overall CVD risk, providing valuable insights into the direct link between smoking exposure and cardiovascular health. Additionally, Ting Lei’s study, which provided insights into the systemic toxicity of smoking exposure, including associations with obesity, bone mineral density, and various diseases, further supports the understanding of the wide-ranging health implications of tobacco exposure [21].

In comparison with the previous studies discussed, including those by Omayma Alshaarawy, Affan Irfan, Ryan Brunetti, and Ting Lei [18–21], our investigation stands out for its new findings regarding the combined effects of smoking with established CVD risk factors. While these prior studies explored various aspects of smoking exposure and its association with specific cardiovascular outcomes, none have specifically examined how smoking interacts with established CVD risk factors to increase overall CVD risk.

Our study revealed that smoking combined with factors such as low HDL-cholesterol, hypertension, central obesity, diabetes, and high triglycerides significantly increases CVD risk, with the combination of multiple risk factors posing the highest risk. This comprehensive analysis underscores the importance of considering the synergistic effects of smoking and established CVD risk factors, offering a new perspective on the relationship between smoking and cardiovascular health. As far as our knowledge extends, no previous study has produced results similar to these, highlighting the novelty and significance of our findings in advancing the understanding of smoking-related CVD risk.

Central obesity, a known risk factor for CVD [13, 19], is often assessed using various indicators such as WHtR, VAI, ABSI, and CI [22]. In our study, we observed significant associations between these indicators and an increased risk of CVD, highlighting their utility in identifying individuals with unfavorable fat distribution patterns that predispose them to CVD [23, 24]. Previous research demonstrates a positive association between central obesity indicators and CVD risk [13, 23, 24]. However, by examining the connection between central obesity and CVD risk in a cohort of smokers, our study adds a unique perspective. This novel approach adds depth to existing knowledge by elucidating the interplay between smoking, central obesity, and CVD risk, contributing to a more comprehensive understanding of cardiovascular health in the context of smoking exposure.

An elevated TyG index has been linked to increased risks of cardiovascular events, including coronary heart disease, myocardial infarction, and stroke [22, 25]. Recognized as an independent risk factor for CVD, the TyG index acts as a marker for insulin resistance, playing a crucial role in the development of metabolic disorders like type 2 diabetes [22, 25]. Some studies suggest that the TyG index may offer better predictive capabilities for CVD risk compared to individual parameters like fasting blood glucose or triglycerides alone [22, 25]. Our study shows strong associations between the Triglyceride-Glucose Index (TyG) and increased risk of CVD in a cohort of smokers. This adds a new perspective to previous research since, to the best of our knowledge, no previous study assessed the TyG index in relation to CVD risk among smokers. By specifically exploring the relationship between the TyG index and CVD risk in smokers, our study introduces a new perspective that was not investigated before.

Our study initially identified a significant association between high PLR and an increased risk of CVD. This finding aligns with previous studies that reported associations between elevated PLR and an increased risk of CVD events, such as myocardial infarction and stroke [26, 27]. These studies also suggested PLR as a potential inflammatory marker and predictor of CVD risk [26, 27]. However, similar to our results, some previous studies reported the loss of significance in the association between PLR and CVD risk after adjusting for multiple factors [26, 28, 29], highlighting the importance of considering various factors when using PLR as a risk indicator for CVD assessment.

Elevated NLR has been linked to an increased risk of cardiac arrhythmia and death in patients with acute coronary syndrome [30]. A study by Bhat et al. demonstrated that a higher NLR is associated with increased mortality among patients with congestive heart failure [30]. Our study’s findings are consistent with those of these previous studies, indicating an elevated NLR’s association with increased CVD risk [29, 30]. Furthermore, a study by Chen et al. suggested that NLR and PLR can predict in-hospital mortality risk in elderly patients with acute myocardial infarction, with NLR showing better predictive ability than PLR [29]. Similarly, our study found that NLR remained significantly associated with an increased risk of CVD even after adjusting for several factors, whereas PLR lost its significance after these adjustments.

Initially, our study identified a significant association between an elevated white blood cell count (WBC) and an increased risk of CVD. However, this association lost statistical significance after careful adjustment for multiple factors. Our findings hinted at a potential link between elevated WBC count and CVD risk. Studies by Barron et al. and Balta et al. emphasized the role of atherosclerosis in increasing the risk of CVD among individuals with elevated WBC counts [31, 32]. Elevated WBC counts are associated with increased inflammation, a critical factor in systemic atherosclerosis [28, 33, 34].

Previous research has demonstrated associations between an increased risk of CVD and various inflammatory markers, including WBC count, neutrophil count, NLR, and PLR [26, 28, 29, 33]. However, it’s essential to consider that the strength of the association between these inflammatory markers and the actual risk of CVD may diminish after adjustments for variables such as age, gender, smoking, and other factors. Our study’s findings align with previous research, as some inflammatory markers, like WBC and PLR, initially showed a significant increase in CVD risk but lost their significance after adjusting for multiple factors.

It’s important to note that the associations between WBC, neutrophil count, PLR, NLR, and CVD risk can be influenced by various factors. While these blood cell counts and ratios provide valuable insights into inflammation and potential CVD risk [26, 29, 30, 32] they are not specific or direct markers of CVD. Multiple factors can influence these blood cell counts and ratios, and their associations with CVD risk may vary depending on the study population, design, and adjustments made for confounding factors [34]. As a result, these blood cell counts and ratios should be considered alongside other risk factors and clinical assessments. Further research is needed to better understand the mechanisms underlying these associations and their interactions with other factors in the development and progression of CVD.

Our study’s uniqueness regarding blood cell count and ratios stems from the nature of our participants, who were specifically assessed for serum cotinine levels, a marker of smoking. Unlike other studies that did not account for smoking exposure, our investigation involved participants with serum cotinine levels revealing a new perspective regarding the use of blood cell count and ratio in CVD risk assessment. To our knowledge, no other study has explored the association between blood cell count and ratios in relation to CVD risk among smokers, highlighting the originality of our research.

In our study, we examined emerging CVD risk indicators, including various blood cell counts and ratios, which have not been thoroughly explored in previous research. We assessed eight of these indicators (WHTR, VAI, ABSI, CI, TyG, NEUT, NLR, and MPV) for their ability to detect CVD risk. These indicators displayed moderate but discerning capabilities in discriminating CVD risk, with AUC values ranging from 0.5368 to 0.7118. This suggests their potential for early CVD detection and risk assessment. To the best of our knowledge, this is the first study to involve all these CVD risk indicators in one study and assess their ability to detect CVD risk.

This study’s strength lies in its comprehensive approach. It examined associations between well-established cardiovascular risks and disease development. It also investigated the impact of combining smoking with individual risks on CVD development and explored the combined effects of smoking with multiple CVD risks. In addition, it scrutinized associations between cardiovascular risk indicators and CVD risk. Lastly, the study assessed combined smoking-indicator risks and analyzed each cardiovascular risk indicator’s detection ability.

## What is new in our study?

Our study of the complex relationship between smoking and the risk of CVD, as measured by blood cotinine levels, has revealed several new findings that advance our understanding of cardiovascular epidemiology.

### Impact of cotinine levels on CVD risk

Our analysis revealed a novel association between cotinine levels and CVD risk. Specifically, the Adjust II Model, which accounted for a wide range of covariates, demonstrated a 25% increase in CVD risk for each unit increase in cotinine levels. Notably, individuals in the highest cotinine quartile (Q4) experienced a 2.33-fold increase in CVD risk, underscoring the potent risk cotinine poses for cardiovascular health.

### Association of traditional risk factors with CVD in smokers

Our study delineates how traditional CVD risk factors—when considered in conjunction with smoking—augment CVD risk. We observed:

- A 1.01-fold increase in CVD risk per unit increase in waist circumference.
- A 1.17-fold increase per unit rise in triglyceride levels.
- There was an inverse relationship with HDL-c, where each unit increase led to a 0.59-fold decrease in CVD risk.
- Hypertension and diabetes mellitus were strongly correlated with elevated CVD risk among smokers, highlighting the compounding effect of smoking on these conditions.

### Synergistic effects of smoking and cardiovascular risk factors

Our findings reveal the synergistic impact of smoking combined with other cardiovascular risk factors, marking a significant advancement in understanding CVD risk dynamics. For example:

- Central obesity in smokers was linked to a 3.04-fold increase in CVD risk.
- Elevated triglyceride levels and reduced HDL-c levels in smokers were associated with a 2.72-fold and 3.82-fold increase in CVD risk, respectively.

### Cumulative risk from multiple risk factors

This study is among the first to quantify the cumulative risk of CVD from smoking in combination with multiple risk factors. Smokers with central obesity, high triglycerides, low HDL-c, and hypertension faced the highest CVD risk, with an odds ratio of 14.18 in the Adjust II model.

### Significance of novel cardiovascular risk indicators

We have identified significant associations between novel cardiovascular risk indicators and CVD risk, especially when considering the combined effects of smoking. This includes:

- A 2.51-fold increase in CVD risk is associated with high WHtR in smokers.
- A notable 3.05-fold increase in CVD risk with elevated VAI levels in smokers.
- A 2.98-fold and 3.28-fold increase in CVD risk for smokers with high ABSI and CI, respectively.
- Elevated TyG-Index, Neutrophil, NLR, and MPV in smokers were all significantly associated with increased CVD risk.

### Limitations of the study

1. While our study adds to the existing body of knowledge, it is not without limitations. NHANES, which collected data through cross-sectional surveys, provides valuable but limited insights due to its inability to establish causality or track changes over time.
2. Our study classified patients into CVD and non CVD groups using self-reported data. Misclassification bias is possible, even though NHANES uses validated questionnaires and cross-verification with medical data. However, the NHANES data’s comprehensiveness and validation reveal population health patterns and risk factors.
3. Since our results have not been replicated in other cohorts, generalizing our findings to other cohorts must be approached with caution.
4. We did not account for ancestry variety in our sample. Further study using ancestry-stratified analyses is needed to determine cardiovascular risk factor differences across ancestral groupings.

## Conclusion

Our cross-sectional study provides insights into the relationship between smoking, various cardiovascular risk factors, and their combined influence on CVD risk. We found a clear dose-response association between serum cotinine levels, a marker of smoking, and CVD risk, emphasizing the detrimental impact of smoking on cardiovascular health. Moreover, our investigation into the combined effects of smoking with well-established CVD risk factors revealed a synergistic increase in CVD risk, particularly when smoking was combined with central obesity, elevated triglycerides, low HDL-cholesterol, and hypertension. Furthermore, our in-depth evaluations of several emerging cardiovascular risk indicators in terms of their ability to detect CVD risk revealed that these emerging markers exhibited moderate discriminatory capabilities. Overall, our study underscores the importance of considering multiple risk factors concurrently in CVD prevention and management and highlights the potential of emerging indicators in enhancing risk assessment.

### Supplementary Information

**Supplementary Material 1.**

**Supplementary Material 2.**

## Data Availability

The data supporting the results of our submitted manuscript are derived from the National Health and Nutrition Examination Survey (NHANES). Due to NHANES data usage policies, the dataset cannot be directly provided or linked. However, it can be independently accessed through the NHANES online portal (https://www.cdc.gov/nchs/nhanes/index.htm). All necessary permissions and ethical considerations regarding the use of NHANES data have been diligently adhered to in our research.
