# Supplementary material for: Investigating the interplay of smoking, cardiovascular risk factors, and overall cardiovascular disease risk: NHANES analysis 2011–2018
Source: BMC Cardiovasc Disord. 2024 Apr 4;24:193. doi: 10.1186/s12872-024-03838-7 (PMC10993506; doi:10.1186/s12872-024-03838-7)
Supplement: Supplementary file 1 — Supplementary Material 1. [file 12872_2024_3838_MOESM1_ESM.pdf]

Relationship between serum Cotinine and CVD risk stratified by gender

Exposure	Non-adjusted<br>OR (95%CI) *P*-val	Adjust I<br>OR (95%CI) *P*-val	Adjust II<br>OR (95%CI) *P*-val
Male			
Log Cotinine	0.99 (0.93, 1.05) 0.7410	1.15 (1.07, 1.24) <0.0001	1.25 (1.15, 1.36) <0.0001
Log Cotinine (quartile)			
Q1	Ref	Ref	Ref
Q2	0.58 (0.40, 0.84) 0.0034	0.71 (0.48, 1.06) 0.0947	0.69 (0.44, 1.08) 0.1050
Q3	0.76 (0.57, 1.02) 0.0682	1.16 (0.83, 1.62) 0.3768	1.54 (1.05, 2.25) 0.0258
Q4	0.72 (0.55, 0.96) 0.0240	1.55 (1.12, 2.14) 0.0083	2.25 (1.53, 3.32) <0.0001
P for trend	0.0640	0.0032	<0.0001
Female			
Log Cotinine	1.12 (1.05, 1.21) 0.0014	1.30 (1.20, 1.40) <0.0001	1.27 (1.15, 1.39) <0.0001
Log Cotinine (quartile)			
Q1	Ref	Ref	Ref
Q2	1.08 (0.76, 1.54) 0.6513	1.28 (0.88, 1.86) 0.1970	1.17 (0.77, 1.78) 0.4525
Q3	0.97 (0.70, 1.35) 0.8610	1.30 (0.91, 1.86) 0.1427	0.86 (0.58, 1.29) 0.4677
Q4	1.55 (1.12, 2.13) 0.0073	3.07 (2.14, 4.41) <0.0001	2.57 (1.71, 3.88) <0.0001
P for trend	0.0325	<0.0001	0.0011

Non-adjusted model adjust for: None
Adjust I model adjust for: Age; Race
Adjust II model adjust for: Age; Race; SBP; DBP; BMI; Waist; HDL-c; TG; LDL-c; TC; HbA1c; FPG; A; ALT; AST; BUN; CR; TB; UA; Alcohol; HTN; DM; CKD; Anti-platelet; β-blockers; ACEI/ARB; Statin; Diuretic; Anticoagulant
