# Supplementary material for: Investigating the interplay of smoking, cardiovascular risk factors, and overall cardiovascular disease risk: NHANES analysis 2011–2018
Source: BMC Cardiovasc Disord. 2024 Apr 4;24:193. doi: 10.1186/s12872-024-03838-7 (PMC10993506; doi:10.1186/s12872-024-03838-7)
Supplement: Supplementary file 2 — Supplementary Material 2. [file 12872_2024_3838_MOESM2_ESM.pdf]

CVD detection metric performance of various CVD risk indicators using receiver operating characteristic (ROC)

	Best threshold	ROC area (AUC)	Specificity	Sensitivity	Accuracy	Positive-LR	Negative-LR	Positive-pv	Negative-pv
WHTR	0.5690	0.6450	0.4459	0.7661	0.4803	1.3826	0.5246	0.1428	0.9405
VAI	1.3420	0.5815	0.5137	0.6147	0.5246	1.2641	0.7500	0.1322	0.9171
ABSI	8.4040	0.7118	0.7192	0.6055	0.7070	2.1562	0.5485	0.2062	0.9380
CI	7.5299	0.7150	0.5373	0.7875	0.5642	1.7019	0.3956	0.1702	0.9545
TyG	7.5961	0.5990	0.4572	0.6835	0.4815	1.2591	0.6923	0.1317	0.9230
NEUT	3.8500	0.6048	0.5618	0.6162	0.5677	1.4063	0.6831	0.1449	0.9239
NLR	2.3333	0.6229	0.7043	0.4893	0.6811	1.6545	0.7252	0.1662	0.9196
MPV	8.1500	0.5368	0.4299	0.6407	0.4526	1.1238	0.8359	0.1193	0.9085
